# Evaluation of a mid-career investigator career development award: Assessing the ability of OppNet K18 awardees to obtain NIH follow-on research funding

**DOI:** 10.1371/journal.pone.0192543

**Published:** 2018-02-13

**Authors:** Cassidy A. Pomeroy-Carter, Sharon R. Williams, Xueying Han, William N. Elwood, Brian L. Zuckerman

**Affiliations:** 1 IDA Science and Technology Policy Institute, Washington, D.C., United States of America; 2 Office of Behavioral and Social Sciences Research, National Institutes of Health, Bethesda, MD, United States of America; Aarhus Universitet, DENMARK

## Abstract

The National Institutes of Health (NIH) K18 award mechanism provides funded opportunities for established investigators to gain knowledge in fields outside of their primary disciplines, but outcomes associated with these awards have not been evaluated to date. NIH’s Basic Behavioral and Social Sciences Opportunity Network (OppNet) is one of the few initiatives that has used this award mechanism. We explored how the unique features of K18 awards affect the ability of recipients to obtain follow-on NIH research funding. We compared outcomes (*ability to obtain follow-on funding* and *interval between receipt of the primary award and receipt of the first follow-on award*) associated with OppNet K18 awards to findings from evaluations of other NIH career development (K) awards, which usually target early-career investigators. We hypothesized that K18 award recipients might be (1) more successful than are other K award recipients in obtaining follow-on NIH research funding due to their career experience or (2) less successful due to the competing demands of other projects. By analyzing follow-on NIH research awards and interview data, we found that OppNet K18 award recipients were at least as successful as were other K award recipients in obtaining follow-on funding and may have been more successful by certain measures. K18 awards produce their outcomes with a lower investment per investigator than do other K awards, suggesting continued or enhanced use of the mechanism.

## Introduction

Scientists who rigorously engage with their own disciplines yet also seek opportunities to integrate approaches of other fields have been identified as catalysts for scientific interdisciplinarity [1]. It has been suggested that many of the research questions of interest to modern society demand an interdisciplinary approach, and the business case for interdisciplinary research has been described in terms of its ability to facilitate (1) access to expertise, instruments, and funds; (2) cross-fertilization among disciplines; (3) the pooling of expertise to solve especially complex problems; and (4) enhanced productivity, among others [2–3]. More specifically, interdisciplinary researchers can stimulate innovative solutions to complex health problems [4]. For over a decade, the National Institutes of Health (NIH) Office of Behavioral and Social Sciences Research (OBSSR) has identified interdisciplinary research as a key component of its strategic vision. OBSSR has recognized that “diverse disciplines, including genetics, neuroscience, computer science, and engineering, are reinvigorating the behavioral and social sciences with novel approaches and methodologies and are cross-pollinating behavioral and social sciences research approaches” [5]. Moreover, interdisciplinary and transdisciplinary approaches help “fully elucidate the complex determinants of health and health-systems challenges” [6]. Investigators may acquire transdisciplinary skills over the course of their careers or as part of their formal training; However, funded opportunities have been limited for mid-career or senior investigators to dedicate time to learn principles and approaches outside of their primary disciplines.

NIH’s K18 award mechanism is designed, in part, to foster the development of such interdisciplinary researchers by providing “support for experienced scientists who wish to broaden their scientific capabilities or to make changes in their research careers by acquiring new research skills or knowledge” [7]. The K18 award mechanism is part of a suite of NIH research career development, or “K” awards. Broadly speaking, these awards are designed to provide support and protected time for intensive, supervised career development experiences [8]. Many of these awards are considered early-career-development awards and are targeted toward recently trained scientists, clinician scientists, or clinicians committed to patient-oriented research (e.g., K01, K08, and K23 award mechanisms). Individuals who obtain these early-career-development grants usually receive three to five years of salary support, during which they are expected to work to establish an independent research career in either laboratory or clinical research.

The K18 award mechanism differs from these more common K award mechanisms in two respects: (1) the K18 award mechanism usually is targeted toward mid-career or senior investigators rather than early-career investigators, and, consequently, (2) the salary and research support is short-term (usually one or two years) and intended to foster a mentored career *enhancement* experience. For K18 awards, career enhancement may include the acquisition of new skills or techniques or the expansion of expertise into new disciplines. The skills and knowledge gained are expected to complement, augment, or redirect the investigator’s existing research program. Individuals who receive K18 awards usually conduct a collaborative research project in a host mentor’s lab and participate in didactic and laboratory-based training activities.

According to data downloaded from NIH’s Research Portfolio Online Reporting Tool, Expenditures and Results (RePORTER), NIH awarded 66 K18 awards between 2006 and 2016. The OBSSR’s Basic Behavioral and Social Sciences Opportunity Network (OppNet) is one of the few NIH initiatives that has used the mechanism, and OppNet K18 awards, which were granted between 2010 and 2014, accounted for 41% of all NIH K18 awards granted between 2006 and 2016 by count (27 of 66 awards). As part of its mission to promote basic behavioral and social science research across NIH, OppNet used the K18 award mechanism to “provide candidates with protected time to achieve a shift in the focus of their research direction in the basic behavioral and social sciences, or to substantially enrich a current basic behavioral and social science research program through the introduction of tools, theories or approaches from another discipline or area of science” [9]. OppNet K18 awards were targeted toward biomedical researchers intending to gain skills in the behavioral and social sciences and behavioral and social science researchers aiming to increase their knowledge and skills in biomedical domains [9].

Perhaps because the K18 award mechanism is used infrequently at NIH, to our knowledge, outcomes associated with the mechanism have not been evaluated to date. This paper reports on an evaluation of NIH’s K18 award mechanism, as implemented by OppNet, to better understand what outcomes flow from the unique features of the award. We selected ability to obtain NIH follow-on research funding related to the K18 award as the primary outcome metric because the development of research skills is a goal of the K18 award mechanism, and subsequent engagement with K18-related topics is one way to evaluate the integration of those capabilities into future research endeavors.

Previous studies have evaluated NIH career development awards and used the ability of awardees to obtain NIH follow-on funding as an outcome metric [10–17]. These studies considered a variety of NIH funding mechanisms, including K01, K07, K08, K11, K22, K23, K25, KL2, and R03 awards. In contrast to the K18 award mechanism, these funding mechanisms are primarily targeted toward early career researchers. Although not a K award mechanism, the R03 small grant program listed among the NIH funding mechanisms examined is similar to the OppNet K18 award mechanism in that it was designed to “encourage new investigators and established investigators interested in refocusing their expertise to apply their skills to behavioral cancer prevention and control research” and included a mentoring component [17].

Using results from these studies as a literature comparison, our study was designed to evaluate how the unique features of the K18 award mechanism identified above may affect the ability of awardees to obtain K-related follow-on funding as compared to other K award recipients. We considered two potential hypotheses with opposite, though not necessarily mutually exclusive, implications: K18 award recipients may be (1) more successful than are other K award recipients in obtaining follow-on NIH research funding due to their career experience or (2) less successful due to the competing demands of other projects.

To investigate these hypotheses, we examined the ability of OppNet K18 award recipients to obtain subsequent NIH research funding and, where possible, compared the results to literature available on similar outcomes in early-career-development K award recipients at NIH. Results from our study may provide insights to the design of future OppNet K18 and other solicitations.

## Methods

### Characteristics of study subjects

Using data downloaded from RePORTER, we determined that between 2010 and 2014, OppNet issued three K18 funding opportunity announcements and awarded 27 K18 grants totaling $2.7 million. These 27 awards ranged widely in focus, but, in accordance with the OppNet K18 program design and goals, the awards were primarily used by biomedical researchers to incorporate social science methodologies (e.g., behavioral economics) into their research; by social scientists to incorporate biomedical approaches (e.g., epigenetics) into their research; by researchers interested in translating their work in animal models to humans; or by investigators interested in creating animal models to study phenomena observed in humans. The abstracts of the 27 awards are provided as a supplementary file (S2 File).

Biographical sketches of K18 investigators (from public sources) were used to identify the demographics of the K18 awardees. Approximately 60% of the awardees were female (16 of 27). Six of the 27 (22%) OppNet K18 awardees held MDs, while the remaining 21 (78%) awardees held PhDs. None of the OppNet K18 award recipients held both an MD and a PhD. Based on degree and department of academic appointment, we classified the awardees as either biomedical/public health researchers or behavioral/social scientists. Two team members made the initial classification, with a third team member, and subject matter expert, adjudicating discrepancies. Sixteen awardees were classified as biomedical/public health researchers, and 11 were classified as behavioral/social scientists. At the time of their awards, 10 of the 27 awardees (37%) held the title of “Professor.” For subsequent analyses, these investigators were classified as “senior investigators.” Most of the remaining awardees held positions as associate or assistant professors, though one awardee held the title of program director and another awardee was listed as PI. These individuals were classified as “mid-career investigators” for subsequent analyses.

### Identification of subsequent NIH research funding

To assess the ability of OppNet K18 awardees to obtain subsequent NIH research funding, we searched RePORTER using each K18 awardee’s name. Any new or competitively renewed grant with an OppNet principal investigator (PI) listed as PI or co-PI and with a project start date subsequent to the K18 award start date was considered subsequent NIH funding for that K18 PI. To focus specifically on research funding, we limited our consideration to NIH research awards. Awards that OppNet K18 investigators received and that were included in our analyses were grants with R01, R21, R34, DP1, DP3, U01, P30, and P50 activity codes. We classified the DP1 NIH Director’s Pioneer Award and the DP3 Type 1 Diabetes Targeted Research Award as R01-equivalent awards. OppNet K18 awardees also received awards through non-NIH and nonresearch mechanisms, including R13, R25, R43, I01, and U48 awards, but we excluded these from our analyses because conference, course-development, and infrastructure development awards fall outside the scope of this project.

### Identification of K18-related follow-on funding

We identified a subset of the subsequent NIH research awards received by K18 awardees as awards related to the K18 award. Subsequent awards were classified as such if they met at least one of three inclusion criteria:

1. *The OppNet PI identified the award as related to the previous K18 work*. We conducted semi-structured interviews with 20 of the 27 OppNet K18 PIs in fall 2016 and spring 2017 (additional details on the interview methodology are provided below). If an interviewee identified a subsequent NIH research award as attributable, at least in part, to the K18 award, we considered that award K18-related follow-on funding.
2. *The subsequent grant application acknowledged the K18 grant*. Using RePORTER, we examined grant applications for NIH research awards received by each K18 investigator subsequent to receipt of their K18 award to identify whether the K18 award was acknowledged in the research description or in the preliminary research sections. If the grant number or K18 award was acknowledged, we considered the award K18-related follow-on funding.
3. *The award was co-acknowledged with the K18 grant on a publication*. Using RePORTER, we identified all publications that acknowledged an OppNet K18 grant as of April 2017. We queried PubMed using the PubMed ID for each of these publications and examined the full list of acknowledged grants. Subsequent NIH research grants that were acknowledged on publications that also acknowledged an OppNet K18 grant were considered K18-related follow-on awards.

Awards were initially classified by two members of the research team. In cases of discrepant classifications, a third member and subject matter expert, examined the classifications and adjudicated discrepancies.

### Calculation of follow-on award timing

For those OppNet K18 awardees who received K18-related follow-on NIH research funding, we examined the interval between receipt of the K18 award and receipt of the first subsequent K18-related follow-on award. Using data derived from RePORTER, we calculated this interval as the number of days between the project start date for the OppNet K18 award and the date of award notice for the K18-related follow-on grant. To focus specifically on the first major research award subsequent to the K18 award, we then repeated this analysis but restricted our consideration to K18-related follow-on R01 or R01-equivalent awards (i.e., DP1 and DP3 awards).

### Statistical comparison of OppNet K18 awardee subgroups

We generated four multivariate generalized linear models (GLMs) to assess the influence of awardee characteristics on (1) ability to obtain K18-related follow-on funding, (2) ability to obtain K18-related R01 or R01-equivalent follow-on funding, (3) number of days between K18 project start date and receipt of K18-related follow-on funding, and (4) number of days between K18 project start date and receipt of K18-related R01 or R01-equivalent follow-on funding. For each GLM, we used a type-III (Wald chi-square) analysis-of-deviance to determine whether awardee characteristics were significant factors in influencing individuals’ abilities to receive follow-on funding or timing to follow-on funding. All analyses were performed using R (version 3.3.2, 2016).

To assess whether an investigator’s ability to obtain K18-related follow-on funding, depended on *career status* at the time of award (senior versus mid-career investigator), *research area* (behavioral or social scientist versus biomedical researcher), *sex*, and *degree type* (MD versus PhD), we used a GLM with a binomial distribution. Similarly, a GLM with a binomial distribution was used to assess whether an investigator’s ability to obtain K18-related R01 or R01-equivalent follow-on funding depended on *career status*, *research area*, *sex*, or *degree type*.

For awardees who received K18-related follow-on funding and those who received K18-related follow-on R01 or R01-equivalent funding, GLMs with Poisson distributions were used to assess whether *career status*, *research area*, and *sex* affected (1) the amount of time (in days) between K18 project start date and receipt of K18-related follow-on funding and (2) the amount of time (in days) between K18 project start and receipt of K18-related follow-on R01 or R01-equivalent funding. We did not include the *degree type* variable in the timing to K18-related follow-on award analyses because of sample size limitations (only one MD received follow-on funding).

### Interviews with OppNet K18 awardees

In addition to our use of interviews to identify follow-on awards, interviews with OppNet K18 awardees provided additional information on outcomes associated with receipt of the K18 award and barriers to K18 award success. Verbal informed consent was obtained from each participant at the start of the interview. Interviews covered three primary topics: (1) participant motivation to apply for the K18 award, (2) activities and outcomes associated with the K18 award, and (3) reflections on the K18 award and suggestions for potential improvements to the OppNet K18 process. The interview guide is attached as a supplementary data file (S3 File).

By consulting notes, transcripts, and audio recordings of interviews, we summarized and organized the results of this qualitative assessment and identified common themes across interviews. Specific quotes were then organized based on themes and used for additional analyses. Initial analysis, coding, and sorting of responses was conducted by a single individual, and other team members who participated in the interviews reviewed the results in order to check validity. Awardee comments provided context and a first-hand perspective that supplemented our analysis.

## Results

### Ability to obtain NIH follow-on funding

As of spring 2017, 16 of the 27 (59%) OppNet K18 awardees had received at least one NIH research award (R21, R34, U01, P30, P50, or R01/R01-equivalent award) subsequent to the award date of the K18. A total of 24 such awards were identified, including 15 R01 or R01-equivalent (i.e., DP1 and DP3 awards) projects awarded to 12 of the 27 OppNet K18 investigators.

Eleven of the 27 K18 awardees (41%) received 13 subsequent awards that built upon their K18 research. Eight of the OppNet K18 awardees (30% of the 27 awardees) received follow-on R01 or R01-equivalent awards (Table 1). These awards included seven R01 awards received by seven separate K18 awardees, one DP1 award (whose recipient did not also receive a follow-on R01 award), and one DP3 award (whose recipient also received a follow-on R01 award). The remaining four of the 13 K18-related follow-on awards included one P30 Research Program Center Core Grant, an individual project as part of a P50 Research Program Specialized Center Grant, and two R21 Research Project Exploratory/Developmental Grants.

**Table 1:** Number of OppNet K18 awardees receiving NIH Follow-on research funding, by year of award.

RFA Year	Number of Awardees	Number of Awardees who Received NIH Follow-On Funding N (%)	Number of Awardees who Received R01/R01-Equivalent NIH Follow-on Funding N (%)	Number of Awardees who Received K18-Related NIH Follow-On Funding N (%)	Number of Awardees who Received K18-Related R01/R01-Equivalent NIH Follow-On Funding N (%)
2010	16	10 (63%)	9 (56%)	6 (38%)	5 (31%)
2011	8	4 (50%)	2 (25%)	3 (38%)	2 (25%)
2014	3	2 (67%)	1 (33%)	2 (67%)	1 (33%)
Total	27	16 (59%)	12 (44%)	11 (41%)	8 (30%)

### Characteristics of OppNet K18 awardees receiving K18-related follow-on funding

Table 2 presents the number of investigators who received K18-related NIH follow-on research funding and the number of investigators who received K18-related R01 or R01-equivalent funding by awardee characteristic (including *career status*, *research area*, *degree type*, and *sex*) alongside the characteristics of the OppNet K18 recipient pool as a whole.

**Table 2:** OppNet K18 recipient pool and OppNet K18 recipients who received follow-on funding by awardee characteristic.

Independent Variable		OppNet K18 Recipient Pool	Investigators who Received K18-Related NIH Follow-on Research Funding	Investigators who Received K18-Related R01 or R01-Equivalent Funding
*Career Status*	Senior Investigator	37% (10/27)	30% (3/10)	20% (2/10)
	Mid-Career Investigator	63% (17/27)	47% (8/17)	35% (6/17)
*Research Area*	Behavioral or Social Scientist	41% (11/27)	45% (5/11)	36% (4/11)
	Biomedical Researcher	59% (16/27)	38% (6/16)	25% (4/16)
*Degree Type*	MD	22% (6/27)	17% (1/6)	17% (1/6)
	PhD	78% (21/27)	48% (10/21)	33% (7/21)
*Sex*	Male	41% (11/27)	27% (3/11)	9% (1/11)
	Female	59% (16/27)	50% (8/16)	44% (7/16)

Analysis-of-deviance results provided no evidence that awardee characteristics (*career status* at the time of award [senior versus mid-career investigator], *research area* [behavioral or social scientist versus biomedical researcher], *degree type* [MD versus PhD], and *sex*) significantly affected the ability of awardees to obtain K18-related NIH follow-on research funding or K18-related follow-on R01 or R01-equivalent funding (*p* > .05 in all cases).

### Follow-on award timing

We calculated the length of time between receipt of the K18 award and receipt of the first NIH K18-related follow-on award for the OppNet K18 awardees. The median time was 3.0 years and ranged from 0.5 years to 5.4 years with a mean of 3.0 years and a standard deviation of 1.6 years. When only R01 and R01-equivalent (i.e., DP1 and DP3) awards were considered, the median was 2.9 years, with a range of 0.6 years to 5.0 years, a mean of 2.9 years, and a standard deviation of 1.3 years.

The median intervals between receipt of the K award and receipt of the (1) first K18-related follow-on award and (2) first K18-related R01 or R01-equivalent award were lower (a) for those K18 recipients classified as senior investigators than for those classified as mid-career investigators, (b) for investigators classified as behavioral and social scientists than for those classified as biomedical investigators, and (c) for female investigators than for male investigators (Table 3).

**Table 3:** Median intervals between K18 award receipt and receipt of K18-related awards (Years) among K18 awardees who received follow-on funding by awardee characteristic.

Independent Variable		Median Interval between Receipt of the K18 Award and Receipt of the First K18-Related Follow-on Award	Median Interval between Receipt of the K18 Award and Receipt of the First K18-Related R01/R01-equivalent Award
*Career Status*	Senior Investigator	1.9	1.2
	Mid-Career Investigator	3.0	3.0
*Research Area*	Behavioral or Social Scientist	3.0	2.4
	Biomedical Researcher	3.4	3.4
*Sex*	Male	5.0	5.0
	Female	2.4	2.9

The analysis-of-deviance type III tests indicated that *career status* at time of award ($\chi_{1}^{2}$ = 340, *p* < .001), *research area* ($\chi_{1}^{2}$ = 207, *p* < .001), and *sex* ($\chi_{1}^{2}$ = 2287, *p* < .001) significantly influenced the interval between receipt of the K18 award and receipt of the first K18-related NIH follow-on research award. Similarly, analysis-of-deviance type III results showed that *career status* ($\chi_{1}^{2}$ = 559, *p* < .001), *research area* ($\chi_{1}^{2}$ = 5.08, *p* = 0.02), and *sex* ($\chi_{1}^{2}$ = 356, *p* < .001) also significantly influenced the amount of time that passed between receipt of the K18 award and receipt of the first K18-related R01 or R01-equivalent award. Specifically, senior investigators received follow-on funding more quickly than did mid-career investigators, behavioral and social scientists received follow-on funding more quickly than did biomedical researchers, and female investigators received follow-on funding more quickly than did male investigators.

We found that *career status* and *research area* were confounding variables for the timing to K18-related follow-on funding analysis. All senior investigators who received K18-related follow-on funding were also behavioral or social science researchers, and most mid-career investigators who received K18-related follow-on funding were biomedical researchers. To assess the severity of multicollinearity between the two variables, we calculated the variance inflation factors (VIFs) for both *career status* and *research area*. VIF values for both were greater than 2.0. Given the small sample size (*N* = 11) for the analysis, VIF values of greater than 2 suggest possible collinearity between the two variables. In addition, the Pearson correlation coefficient also indicated significant correlation between the *career status* and *research area* variables (*p =* 0.02). The influence of the senior status of awardees who received K18-related follow-on funding, therefore, could not be distinguished from the influence of their areas of research in this analysis. There was no evidence that *career status* and *research area* were confounding variables for the timing to K18-related follow-on R01 or R01-equivalent funding analysis.

### K18 interviews

In interviews, OppNet K18 awardees spoke highly of the K18 award mechanism and attributed a diverse range of positive outcomes to their K18 awards. These outcomes ranged from receipt of follow-on funding and publications to career advancement and invitations to establish new research programs and centers. More broadly, interviewees credited their awards with enhancing their professional recognition and broadening their research scope and ability to work in interdisciplinary settings.

Many interviewees indicated that their K18 awards led directly to follow-on projects. For instance, one interviewee identified multiple lines of research that “would have been impossible to do had [he] not had the K18 experience.” In other cases, however, the career and research trajectories of awardees inhibited their ability to pursue additional K18-related projects or to do so in a timely manner. One awardee, for example, indicated that “in order for [her] to be a PI on an NIH grant or a lead researcher on a grant, [she] would have had to have dropped everything else [she] was doing.” In another instance, an interviewee noted delayed outcomes from his award because “there was so much data, and no one [had] ever put the data together before. [They] had to essentially invent methods to be able to look at those data in combination”.

Interviewees were also asked to offer suggestions for improvements to the OppNet K18 program. Notable among these is the statement that “Necessarily, short awards are not going to be very efficient for the goal of training. They should be a little longer.” Awardees suggested that a longer period would have improved their ability to achieve the goals outlined in their grant applications.

## Discussion

Our results indicate that K18 award recipients are equally likely, and may be more likely, to receive any NIH follow-on research funding and to receive R01 or R01-equivalent follow-on funding than are recipients of other K awards, as reported in the literature. We found that 59% of OppNet K18 awardees received NIH follow-on funding and that 44% received R01 or R01-equivalent follow-on funding. Among the literature we examined, three studies assessed the ability of subjects to obtain any NIH follow-on research funding; Values reported in those studies for percentage of K award recipients obtaining any follow-on funding ranged from 36% to 59%, placing our finding on the upper end of this range [14, 16–17]. Similarly, our finding that 44% of OppNet K18 award recipients obtained R01 or R01-equivalent follow-on awards lies on the upper end of the range reported in the literature for other K award recipients: a total of seven studies we examined evaluated the success of researchers in obtaining subsequent major NIH research awards (i.e., R01 Research Project Grants or R01-equivalent awards) and reported results ranging from 14% to 45% [10–13, 15–17].

The percentage of OppNet K18 awardees (1) receiving any *K18-related* follow-on NIH research funding and (2) receiving R01 or R01-equivalent *K18-related* follow-on funding in our study are also in accordance with outcomes reported in the literature for other K award recipients. We assumed that, for early-career-development K award recipients, funding subsequent to the K award was related to the K award itself. The same could not be assumed of K18 award recipients, who were established investigators with potentially competing lines of research. In many cases, interviews confirmed that the K18 award was critical to the development of follow-on projects, though in other cases, awardees indicated that their career and research trajectories inhibited their ability to pursue additional K18-related projects. We compared OppNet investigators’ abilities to obtain K18-related follow-on NIH research funding against the literature’s report of other K award recipients’ abilities to obtain any follow-on NIH research funding (the same outcomes as reported in the previous paragraph). Our findings that 41% of OppNet K18 awardees received K18-related follow-on funding and that 30% of OppNet K18 awardees received K18-related R01 or R01-equivalent follow-on funding indicate that OppNet K18 awardees were neither more nor less likely than other K award recipients to receive K-related, NIH follow-on funding.

We hypothesized that the mid-career or senior-investigator status of K18 awardees might confer an advantage during the application process for subsequent K-related funding that early-stage-investigator K award recipients do not enjoy. Specifically, factors such as significant prior experience with the NIH funding process and application writing; prior establishment of professional networks, publication record, scientific reputation, and institutional support; and demonstrated commitment to a scientific career may be associated with increased success in obtaining follow-on funding. Early-stage-investigator K award recipients may lack the experience required to accrue these advantages, potentially influencing their ability to obtain follow-on funding. At the same time, we also recognized the possibility that the early-stage-investigator status of early-stage-investigator K award recipients could confer an advantage during the application process for subsequent funding attributable to the K award that K18 awardees do not enjoy. During NIH’s review process, early stage investigators, defined by NIH as investigators who previously have not competed successfully as PIs for a substantial NIH independent research award, receive special consideration. In addition, research supported by career development awards may be used as preliminary data for subsequent awards. Because early stage investigators generally lack other sources of preliminary data, their first major award subsequent to receipt of their K award can be assumed to build upon the foundation of the K award and can therefore be considered K-related follow-on funding. The validity of this assumption is compounded by the fact that early-career K awards are intended to fund a minimum of 75% of the investigator’s time, typically over a period of three to five years (e.g., [18]), leaving little time to generate preliminary data through non-K award research. K18 awardees, however, receive funding for 25–50% of their time over a one-year period and are likely to have other sources of preliminary data, so subsequent awards cannot necessarily be assumed to be related to their K18 research. The competing demands of other lines of research, and the ability to pursue those avenues using other (non-K18-related) preliminary data, may delay subsequent K18-related grant submissions or diminish the motivation of K18 awardees to pursue K18-related follow-on research. However, this study found no evidence that either group of awardees (OppNet K18 award recipients or recipients of other K awards as reported in the literature) outperformed the other group in terms of ability to obtain K-related, NIH follow-on funding, and therefore no evidence of the dominance of either of our hypotheses.

Results from our study do suggest that K18 award recipients may receive their K-related follow-on awards faster than do other K award recipients. We examined four studies that analyzed time between receipt of a K award and receipt of the first R01 or R01-equivalent award as an outcome metric; These studies found median intervals ranging from 4.0 to 5.6 years [12–15]. While it has been NIH policy since 2004 for K awardees only to be able to receive other independent funding in the last two years of their award [19], several of the previous studies [12, 14] rely on data from earlier years, and one more recent study measures instead from the end of the K award to first R01 [15]. We found that the median length of time between receipt of the K18 award and receipt of the first K18-related follow-on award for OppNet K18 awardees was 3.0 years for any NIH K18-related follow-on award and 2.9 years for K18-related R01 and R01-equivalent awards, considerably lower than intervals observed in other studies. This relatively short interval for K18 awardees is arguably briefer than might be expected based on interview comments regarding the interdisciplinary and exploratory nature of the work conducted by many K18 awardees.

Review of our stratified data indicates that the smaller interval between receipt of the K award and receipt of follow-on funding for K18 awardees as compared to early-career-development K award recipients may be explained by the more senior status of the K18 awardees: even within our study, the median intervals between receipt of the K award and receipt of the (1) first K18-related follow-on award and (2) first K18-related R01 or R01-equivalent award were significantly lower for those K18 award recipients classified as senior investigators than for those classified as mid-career investigators. However, the *career status* variable confounded with the *research area* variable in the first of these two analyses, making it impossible to distinguish between their individual effects.

Our results indicate that K18 award recipients are at least as successful as are other K award recipients in obtaining follow-on funding and may be more successful in certain measures. K18 awards may produce these outcomes, moreover, with a lower investment from NIH per investigator than do other NIH K awards, because K18 awards fund 25–50% of an investigator’s time over a single year, whereas other K awards provide funding for a minimum of 75% of the investigator’s time over a period of three to five years.

This finding suggests that enhanced use of the K18 approach may be a cost-effective way to boost the research productivity of basic behavioral and social science researchers through an interdisciplinary mentorship award. That said, it should be noted that K18 awards cost NIH less per investigator than do other K awards in part because the K18 award provides fewer years of support to recipients. During interviews with OppNet K18 awardees, the brevity of the period of K18 award support was identified frequently as an aspect of the award in need of improvement. Future use of the K18 award mechanism may incorporate longer performance periods coupled with a subsequent evaluation of whether extended support tends to enhance positive outcomes associated with the award or merely increases the cost to NIH without providing additional benefits beyond those observed for K18 awardees with shorter periods of performance. Longer performance periods also may be more appropriate for investigators to add more complex or novel expertise to their respective skillsets.

While career development for early career investigators has been examined, there are fewer mechanisms supporting mid-career career development and therefore less information on career development during this career stage. Our study provides evidence that, in at least one case, career development support for mid-career researchers can deliver comparable, and possibly even superior, outcomes as compared to support for early career investigators. Future research might examine the mid-career period generally, as well as the potential policy benefits of supporting the career development of mid-career researchers specifically.

We note that in our study we had limited ability to detect differences between subgroups given the small number of funded OppNet K18 awardees (N = 27) and the presence of confounding variables (*career status* and *research area*). Moreover, it was not feasible to identify an appropriate comparison group, though to the extent possible, K18 award outcome data were compared to outcomes associated with other NIH K award mechanisms as reported in the published literature. We further recognize the limitations associated with using interviews, acknowledgments of grants on publications, and acknowledgments of grants on subsequent grant applications to determine the relatedness of awards: investigators may over-attribute subsequent awards to their K18 grants or may forget to convey relevant information during interviews. Similarly, investigators may over-acknowledge or under-acknowledge grants in subsequent grants and publications. Finally, we note that our work is time-censored and that as time passes OppNet K18 awardees may produce additional K18-related publications that we were unable to account for in our analysis. Despite these limitations, results from this study may provide insights to the design of future OppNet K18 and other solicitations.

As stated earlier, NIH has longstanding efforts to train new investigators and to facilitate new and early stage investigators to obtain larger-scale research (i.e., R01) project grants. More recently, the agency announced its Next Generation Researchers Initiative to help early- and mid-career investigators sustain independent research careers beyond initial R01 or equivalent projects [20]. Though our sample size limited the scope of our conclusions, our finding that K18 award recipients may receive subsequent project-related follow-on funding more quickly than do other K awardees suggests that K18 awards can be a cost-effective way to increase support for mid-career investigators; to facilitate the growth of transdisciplinary scientists; and to increase opportunities for behavioral, social, and biomedical mid-career researchers to receive additional R01 awards.

## Supporting information

S1 File Dataset. (XLSX)

S2 File OppNet K18 award abstracts. (CSV)

S3 File Interview guide. (DOCX)
