# Supplementary material for: Evaluation of a mid-career investigator career development award: Assessing the ability of OppNet K18 awardees to obtain NIH follow-on research funding
Source: PLoS One. 2018 Feb 13;13(2):e0192543. doi: 10.1371/journal.pone.0192543 (PMC5810999; doi:10.1371/journal.pone.0192543)
Supplement: S3 File — (DOCX) [file pone.0192543.s003.docx]

# OppNet – K18 PI Interview Guide

### Introduction of Personnel

### Introduction and Informed Consent

### Project Overview

### Introduction

- Date:
- PI Name:
- What is your academic background?
- What is the academic department in which you are currently employed?

### Motivations and Rationales for Proposal Submission

For this first set of questions, we are interested in understanding what led you to pursue a mentored research opportunity in the basic behavioral and social sciences and submit an OppNet K18 proposal.

- What is your general area of research?
- Please tell us about your OppNet K18 research.
- How was your OppNet K18 award different from your previous research?
- How did you hear about the OppNet K18 Request for Applications (RFA)?
- Why did you decide to pursue this K18 award?
- How did you decide to collaborate with your K18 mentor?
- How would you describe your collaborative relationship with your mentor during your OppNet award? (i.e. A mentor/mentee relationship; peer relationship sharing interdisciplinary methods and viewpoints)
- How did you design the career development plan?
  - What types of training activities were included in your OppNet proposal?
  - How did these activities influence your research on your OppNet award?

### Collaboration and Interdisciplinarity

- What types of new collaborations/relationships formed during and as a result of your award?
  - Were these collaborations interdisciplinary?
- As compared to your previous research, was your K18 award different in terms of collaborativeness and interdisciplinarity?
- Have you continued these collaborations after your OppNet K18 award? How?

### Outcomes

- What skills have you gained as a result of your OppNet K18 award?
  - Were they different than what was proposed at the outset of the award?
- What shifts in research understanding or perspective have occurred as a result of your OppNet K18 award?
- Have you continued to use the skills and perspectives you have gained in your K18 award for subsequent research?
  - If so, how?
- Has your K18 award influenced your career trajectory?
  - If so, how?

### Final Questions

- Without the OppNet K18 RFA, do you believe you would have been able to pursue similar mentored research elsewhere at NIH?
- Do you have any questions related to this interview or the overall OppNet evaluation?

### Thank You
